# A Bearing Fault Diagnosis Method Based on Weighted Differential Time–Frequency Features and a Dual-Branch Interactive Fusion Network

**DOI:** 10.3390/s26144507

**Published:** 2026-07-15

**Authors:** Bing Wang, Yushu Lai, Zhen Li, Jiajun Jiang, Baochuan Tan

**Affiliations:** School of Mechanical Engineering, Chongqing Sanxia University of Science and Technology, Chongqing 404100, China; wangbing@stumail.sanxiau.edu.cn (B.W.); truth2310@163.com (Z.L.); jjj965678279@163.com (J.J.); 15340396338@163.com (B.T.)

**Keywords:** bearing fault diagnosis, variable operating conditions, few-shot settings, time-frequency feature fusion, multi-scale wide-kernel convolution, Swin Transformer, interactive feature fusion

## Abstract

Existing methods often have difficulty fully characterizing fault information using a single input feature, and the interaction and fusion between local fault impulse features and contextual relationships remain insufficient, which limits diagnostic performance under variable operating conditions and few-shot settings. To address these issues, this article proposes a bearing fault diagnosis method based on weighted differential time–frequency features (WDF) and a dual-branch interactive fusion network. First, first-order differencing is used to enhance local transient information, and the fast Fourier transform (FFT) is employed to extract frequency-domain features. The two feature streams are then weighted and stacked to form WDF as the model input. Then, a multi-scale wide-kernel deep convolutional neural network (MS-WDCNN) branch is designed to extract multi-scale local fault impulse features, while a Swin Transformer branch is introduced to model contextual relationships in two-dimensional feature representations. Subsequently, a Feature Interaction Module (FIM) is proposed to achieve bidirectional interaction and complementary fusion between the two branches, thereby enhancing the discriminability of fault features. Experiments on two public bearing datasets demonstrated that the proposed method achieved average accuracies of 99.45% and 96.12% across eight CWRU tasks and six HUST tasks under variable operating conditions, respectively. In further few-shot experiments, the number of training samples per class was set to 5, 7, 10, 15, and 20, while the test set remained unchanged. Under the most challenging five-shot setting, the proposed method achieved average accuracies of 89.61% and 74.73% on the CWRU and HUST tasks, respectively, further demonstrating its effectiveness under variable operating conditions and few-shot settings.

## 1. Introduction

Rolling bearings are critical supporting components in rotating machinery, and their operating conditions directly affect equipment safety, system stability, and service life. Localized defects in bearing components, such as inner-race, outer-race, and rolling-element defects, usually manifest as short-duration vibration impulses and abnormal energy transmission. These local degradations are not only typical bearing fault modes but also important indicators of equipment health in mechanical structural health monitoring, condition-based maintenance, and intelligent operation and maintenance. If such localized defects cannot be identified at an early stage, they may further evolve into performance degradation, unplanned downtime, or even safety accidents [1,2]. With the development of industrial equipment toward higher speed, greater complexity, and increased intelligence, bearing fault diagnosis requires not only high recognition accuracy, but also the ability to adapt to complex operating conditions such as varying loads, varying speeds, and noise interference [3]. Meanwhile, fault samples are difficult to obtain in practical engineering scenarios, and limited samples further increase the difficulty of model training and generalization [4]. Therefore, developing highly reliable bearing fault diagnosis methods for variable operating conditions and few-shot settings is of great significance for vibration-sensor-based mechanical SHM [5].

Representative signal processing and time–frequency analysis methods are often used to enhance fault-related information in vibration signals. Existing studies have combined short-time Fourier transform (STFT), continuous wavelet transform (CWT), Hilbert–Huang transform (HHT), empirical mode decomposition (EMD), variational mode decomposition (VMD), and fast Fourier transform (FFT) with deep learning models to construct time–frequency maps, decompose fault components, or extract frequency-domain features, thereby improving bearing fault diagnosis performance [6,7,8,9,10,11]. These methods provide richer fault information for deep models. However, STFT is constrained by a fixed window, whereas CWT, HHT, EMD, and VMD usually depend on parameter selection or decomposition procedures and thus have relatively high computational complexity. Moreover, these methods remain insufficient in simultaneously enhancing local transient information and frequency-domain discriminative information. Therefore, constructing more discriminative input features with relatively low computational complexity remains an important issue in bearing fault diagnosis.

With the improvement in computing capability, deep learning-based data-driven methods have become an important research direction for bearing fault diagnosis [12]. Among them, CNNs and their variants are widely used for fault feature extraction from vibration signals because of their strong local feature learning ability. The WDCNN proposed by Zhang et al. [13] uses a wide kernel in the first convolutional layer to directly process raw vibration signals, which enables effective capture of local fault impulse features. Subsequently, Zhang et al. [14] further investigated the diagnostic performance of deep convolutional networks under noisy environments and different working loads, demonstrating the robustness of wide-kernel convolutional structures under complex operating conditions. Shen et al. [15] combined an attention mechanism with a multi-scale convolutional neural network to stably extract bearing fault feature information. Song et al. [16] extracted high-dimensional features using CNN and combined BiLSTM to model temporal correlations for bearing fault diagnosis under multiple operating conditions and limited samples. Kumar et al. [17] adopted multi-size wide kernels to capture a wider range of fault patterns. Hu et al. [18] combined multi-scale CNN with multiple attention mechanisms to strengthen the extraction of key fault features. Wang et al. [19] introduced a damage-dynamics CNN to enhance model interpretability while maintaining high classification accuracy. However, many existing multi-scale CNNs mainly rely on small-scale kernels or shallow multi-branch convolution to enlarge the receptive field, and lack targeted mechanisms for modeling fast transient impulse variations under variable operating conditions. Under variable speed and load conditions, the repetition interval, impact amplitude, and spectral distribution of localized fault impulses change significantly, making fault impulses easily coupled with background vibration and condition-related components. This makes existing multi-scale CNN methods prone to extracting mixed vibration responses, rather than stably isolating representative fast transient impulse features across different operating conditions. Therefore, a more targeted multi-scale local feature extraction branch is still needed to capture fault impulse information at different temporal scales and enhance the extraction ability of local fault impulse features.

The Swin Transformer provides a feasible solution for alleviating the limitations of CNNs in contextual relationship modeling. The Swin Transformer proposed by Liu et al. [20] uses window self-attention and a shifted-window mechanism to realize local-window modeling and cross-window information interaction. Chen et al. [21] proposed MCSwin-T, which combines a multi-channel calibration mechanism with shifted-window attention for few-shot fault diagnosis under sharp speed variation. Wang et al. [22] designed a Transfer Residual Swin Transformer to enhance rolling bearing feature learning through a residual window-attention structure. Yan et al. [23] combined the generalized S-transform with Swin Transformer to improve the time–frequency representation of non-stationary bearing signals. Sun et al. [24] integrated Swin Transformer with multi-scale convolution in parallel, significantly improving diagnostic performance.

Furthermore, realizing effective interaction between features from different branches through a specialized fusion module is a key issue in dual-branch architecture design. Chen et al. [25] proposed a parallel CNN-Transformer model for bearing fault diagnosis, which fuses complementary local and global features to improve fault representation and diagnostic performance. Li et al. [26] proposed Dconformer, which combines convolution with Transformer to enhance bearing fault feature extraction. Song et al. [27] designed a lightweight CNN-Transformer hybrid model for real-time diagnosis of abrupt and incipient faults in IMUs. Meng et al. [28] proposed Swin TransCNN, which hierarchically integrates complementary information from CNN and Swin Transformer branches through feature fusion blocks. Zhang et al. [29] proposed FGNet, which uses a global–local feature fusion module to aggregate multidomain features from CNN and frequency-domain-guided Swin Transformer and enhance different feature representations. These studies demonstrate that fusion mechanisms contribute to stronger feature representation. However, existing methods still have insufficient inter-branch information interaction and complementary feature utilization, limiting the discriminability of fused features.

To address the above issues, this article proposes a bearing fault diagnosis method based on weighted differential time–frequency features and a dual-branch interactive fusion network. By enhancing input feature representation, jointly modeling local impact information and contextual relationships, and strengthening information interaction between branches, the proposed method improves the discriminability of fault features and the generalization performance of the model. The main contributions of this article are summarized as follows:

1. A weighted differential time–frequency feature construction method is proposed. By integrating differential time-domain features and FFT-based frequency-domain features, the proposed WDF representation enhances the discriminability of input fault information.
2. A dual-branch feature extraction network composed of a multi-scale wide-kernel deep convolutional neural network (MS-WDCNN) and a Swin Transformer is constructed, enabling parallel learning of multi-scale local fault impulse information and contextual information.
3. A Feature Interaction Module (FIM) is designed to realize bidirectional information interaction and complementary fusion between dual-branch features, thereby improving the discriminability of fused features.

The remainder of this article is organized as follows: Section 2 introduces the related theoretical background. Section 3 describes the proposed method in detail. Section 4 presents the experimental results and analysis. Section 5 presents the discussion. Section 6 concludes this article.

## 2. Theoretical Background

### 2.1. Fast Fourier Transform

The fast Fourier transform (FFT) is an efficient implementation of the discrete Fourier transform (DFT), which maps a discrete time-domain signal into the frequency-domain space for analyzing the frequency composition of a signal. For vibration signals, the frequency-domain representation can reflect the amplitude distribution of different frequency components and provide a basis for subsequent feature extraction. The FFT is expressed as follows:

(1) $$X(k)=\sum_{n=0}^{N-1}x(n)e^{-j\frac{2\pi }{N}kn}$$

where *x*(*n*) denotes the discrete time-domain signal, *X*(*k*) denotes the corresponding frequency-domain spectrum, *N* is the total number of sampling points, *k* is the frequency index, and *j* denotes the imaginary unit.

### 2.2. Wide First-Layer Kernel Deep Convolutional Neural Network

The wide first-layer kernel deep convolutional neural network (WDCNN) is a convolutional feature extraction method for one-dimensional vibration signals. Its main characteristic is the use of a large convolution kernel in the first layer. Compared with ordinary small kernels, the wide kernel in the first layer expands the receptive field in the low-level feature extraction stage, enabling the network to capture local response information over a longer time range from raw vibration signals, which is beneficial for extracting fault impulse features. The one-dimensional convolution operation can be expressed as follows:

(2) $$y_{j}=\sigma \left(\sum_{i=1}^{C}x_{i}*w_{ij}+b_{j}\right)$$

where $x_{i}$ denotes the i-th input channel, $w_{ij}$ denotes the convolution kernel between the i-th input channel and the j-th output channel, $b_{j}$ is the bias term, ∗ denotes the convolution operation, σ(⋅) is the activation function, and C is the number of input channels.

### 2.3. Swin Transformer

**Figure 1:**
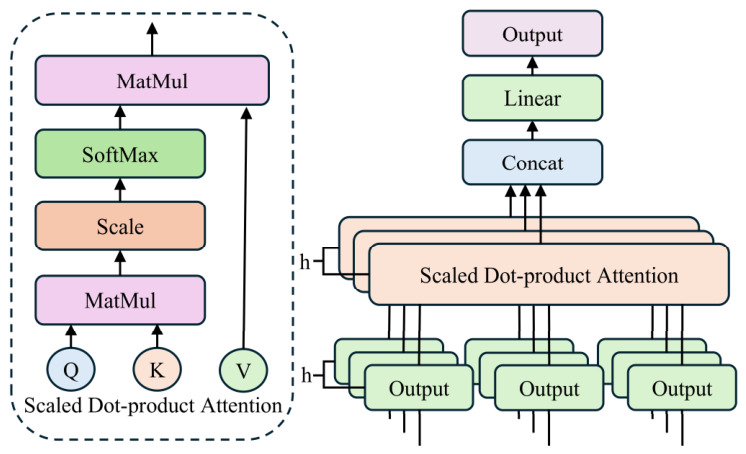
MSA mechanism.

Swin Transformer performs feature modeling based on the multi-head self-attention (MSA) mechanism. MSA calculates feature correlations using the query matrix *Q*, key matrix *K*, and value matrix *V*. A single attention head adopts scaled dot-product attention, which is defined as follows:

(3) $$\mathrm{Attention}\left(Q,K,V\right)=\mathrm{Softmax}\left(\frac{QK^{T}}{\sqrt{d_{k}}}\right)V$$

where *Q*, *K*, and *V* denote the query, key, and value matrices, respectively, *d*_k_ denotes the dimensionality of the query and key vectors in each attention head, and Softmax(·) denotes the softmax normalization function. The above equation describes the calculation process of single-head scaled dot-product attention. In MSA, *h* attention heads perform scaled dot-product attention in parallel and learn feature associations in different feature subspaces, thereby enhancing feature representation. The structure is shown in Figure 1.

## 3. Proposed Method

**Figure 2:**
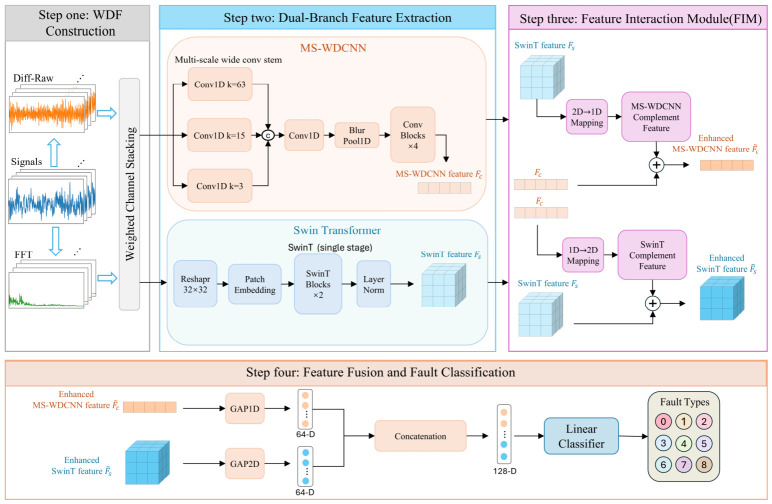
Overall framework of the proposed method.

This section introduces the proposed bearing fault diagnosis method. The method aims to improve the feature representation capability and diagnostic performance of bearing fault diagnosis models under variable operating conditions and few-shot settings. As shown in Figure 2, the overall framework of the proposed method mainly consists of the following four steps.

Step 1: Front-End Feature Construction.

The original vibration signals are processed by differencing and FFT transformation, respectively, to obtain differential features that highlight local transient information and spectral features that characterize frequency-domain distribution information. Then, the two types of features are weighted and stacked along the channel dimension to construct weighted differential time–frequency features (WDF), providing a more discriminative input representation for the subsequent network.

Step 2: Dual-Branch Feature Extraction.

WDF is fed into the feature extraction network. The MS-WDCNN branch uses multi-scale wide convolution to extract local fault impulse information under different receptive fields, while the Swin Transformer branch models contextual relationships through the window-based self-attention mechanism and extracts context-aware features.

Step 3: Feature Interaction and Fusion.

The Feature Interaction Module (FIM) performs bidirectional information interaction between the two branches, enabling local fault impulse information and contextual features to complement each other, thereby enhancing the representation ability of the fused features.

Step 4: Fault Classification.

The interacted and fused features are fed into the classifier to output the health state or fault category of the bearing. Through the above steps, the proposed method can improve the accuracy and stability of bearing fault diagnosis under variable operating conditions and few-shot settings.

### 3.1. Construction of Weighted Differential Time–Frequency Features

To enhance the representation capability of local abrupt variations and frequency-domain fault features in vibration signals, this article constructs weighted differential time–frequency features (WDF) as the model input. Let the raw vibration sample be denoted as follows:

(4) $$x=\left[x_{0},x_{1},\ldots ,x_{L-1}\right]$$

where L=1024. First, a first-order difference operation is applied to the raw signal to highlight local abrupt variations and fault impulse features in vibration signals:

(5) $$d_{i}=x_{i}-x_{i-1},i=1,2,\ldots ,L-1$$

where $d_{0}=0$. Then, the differential signal is standardized sample by sample:

(6) $${\hat{d}}_{i}=\frac{d_{i}-\mu_{d}}{\sigma_{d}+\varepsilon }$$

where $\mu_{d}$ and $\sigma_{d}$ denote the mean and standard deviation of the differential signal, respectively, and ε is a small constant used to avoid a zero denominator.

This sample-wise standardization reduces the effects of operating-condition variations, signal-amplitude differences, and energy fluctuations across samples, thereby giving the differential features a more stable statistical distribution within each sample. It also emphasizes relative local variations in the differential sequence and improves training stability.

Meanwhile, FFT is performed on the raw vibration signal, and the single-sided amplitude spectrum is extracted to obtain frequency-domain fault features.

Let the length of the extracted single-sided amplitude spectrum be denoted by K. The spectral feature reflects the amplitude distribution of different frequency components and provides complementary information to the differential feature, which emphasizes local transient variations. Then, logarithmic compression and standardization are applied to the spectral amplitudes to reduce the dynamic range of amplitudes and improve the stability of frequency-domain features:

(7) $${\hat{s}}_{k}=\frac{log(1+|X_{k}|)-\mu_{s}}{\sigma_{s}+\varepsilon }$$

where $|X_{k}|$ denotes the spectral amplitude after FFT, and $\mu_{s}$ and $\sigma_{s}$ denote the mean and standard deviation of the spectral features, respectively.

Since the standardized differential feature $\hat{d}$ has a length of L, whereas the standardized single-sided spectral feature $\hat{s}$ has a length of K, the two feature streams have inconsistent sequence lengths and cannot be directly stacked along the channel dimension. To construct a unified two-channel input, one-dimensional linear interpolation is adopted to align the length of the spectral feature. Specifically, $\hat{s}$ is regarded as a single-sided spectral amplitude sequence arranged from low to high frequencies and is interpolated from length K to length L, generating the length-aligned frequency-domain feature:

(8) $$\tilde{s}={Interp}_{K\to L}(\hat{s})$$

where ${Interp}_{K\to L}(\cdot )$ denotes one-dimensional linear interpolation from length K to length L, and $\tilde{s}$ denotes the length-aligned frequency-domain feature. During interpolation, the feature values at newly generated positions are estimated from adjacent spectral amplitudes according to a linear relationship, thereby filling the intermediate positions between the original spectral points. This operation is performed only to unify the sequence lengths of the differential and spectral features, thus satisfying the dimensional requirements for subsequent channel stacking and network input. Meanwhile, the interpolation process preserves the original low-to-high frequency ordering of the single-sided spectrum without changing its fundamental interpretation as a frequency-domain amplitude distribution.

**Figure 3:**
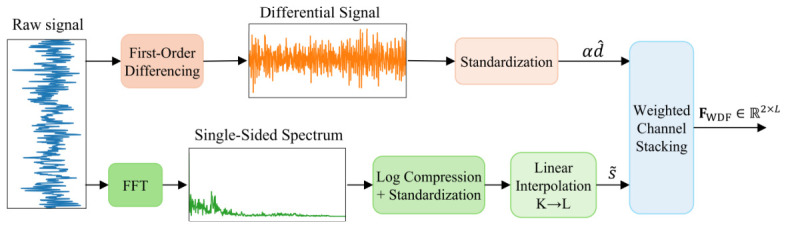
Construction process of WDF.

After the length alignment of the differential and spectral features, the two feature streams are weighted and stacked along the channel dimension to obtain the weighted differential time–frequency features:

(9) $$F_{WDF}=\left[\begin{array}{c} \alpha \hat{d}\\ \hat{s} \end{array}\right],\alpha =0.5$$

where $\hat{d}$ denotes the standardized differential time-domain feature, $\tilde{s}$ denotes the frequency-domain amplitude feature after logarithmic compression, standardization, and interpolation, and α is the weighting coefficient of the differential time-domain feature. Before stacking, both the differential and spectral features are standardized so that they are placed on comparable statistical scales. The weighting coefficient α is used to adjust the relative contribution of the differential channel in WDF. When α is relatively small, the local transient information may not be sufficiently emphasized; when α is excessively large, the differential channel may dominate, thereby weakening the complementary role of the spectral feature. Therefore, α is set to 0.50 in this article to balance local impulse information enhancement and frequency-domain discriminative information preservation. The sensitivity analysis in Section 4.1.5 further verifies the rationality of this setting. After standardization and length alignment, the two feature streams are transformed into a two-channel representation with a consistent sequence length and comparable numerical scales, providing a unified input for the subsequent two feature extraction branches. Specifically, the MS-WDCNN branch extracts local fault impulse information over different temporal ranges through one-dimensional multiscale convolution, whereas the Swin Transformer branch models contextual relationships using window-based self-attention after two-dimensional reconstruction. The construction process of WDF is shown in Figure 3.

### 3.2. MS-WDCNN Branch

**Figure 4:**
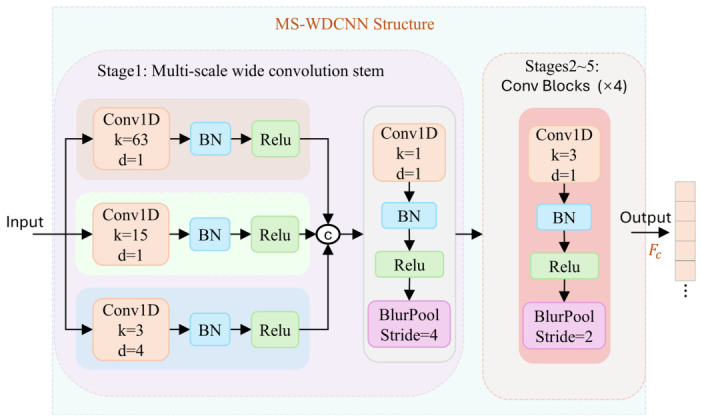
Structure of the MS-WDCNN branch.

To address the limited adaptability of the single wide convolution kernel in the first layer of conventional WDCNN to fault features at different scales, this article constructs a multi-scale wide-kernel deep convolutional neural network (MS-WDCNN) branch. The standardized and length-aligned WDF is directly fed into this branch, allowing one-dimensional convolutions at different scales to jointly process differential and spectral information with a consistent sequence length and comparable numerical scales, thereby reducing the influence of inter-channel scale differences on local feature extraction. This branch consists of five convolutional stages. The first level is a multi-scale wide-kernel convolution stem, which uses parallel large-kernel convolution, medium-kernel convolution, and dilated convolution to enlarge receptive fields at different ranges and extract local fault impulse responses at multiple temporal scales. The following four levels are conventional one-dimensional convolutional blocks, which are used to further extract deep local features. The structure of this branch is shown in Figure 4.

Specifically, the input feature $X\in R^{C\times L}$ is first fed into the multi-scale wide-kernel convolution stem, where C denotes the number of input channels and L denotes the signal length. This layer consists of three parallel convolutional branches:

(10) $$F_{1}=\phi \left(BN\left(Conv_{63}(X)\right)\right)$$

(11) $$F_{2}=\phi \left(BN\left(Conv_{15}(X)\right)\right)$$

(12) $$F_{3}=\phi \left(BN\left(Conv_{3,d=4}(X)\right)\right)$$

where $Conv_{63}(\cdot )$ and $Conv_{15}(\cdot )$ denote one-dimensional convolutions with kernel sizes of 63 and 15, respectively. $Conv_{3,d=4}(\cdot )$ denotes a one-dimensional dilated convolution with a kernel size of 3 and a dilation rate of 4. BN(⋅) denotes batch normalization, and ϕ(⋅) denotes the ReLU activation function.

Then, the features from the three scales are concatenated along the channel dimension and fused by a 1 × 1 Conv1D layer to integrate multi-scale information:

(13) $$F_{m}=\phi \left(BN\left(Conv_{1}\left(\left[F_{1},F_{2},F_{3}\right]\right)\right)\right)$$

where [ · ] denotes channel-wise concatenation, and $F_{m}$ denotes the multi-scale fused feature. To reduce the influence of high-frequency aliasing during downsampling, the fused feature is further processed by BlurPool1D for low-pass filtering and downsampling:

(14) $$F_{d}=BlurPool1D\left(F_{m}\right)$$

where $F_{d}$ denotes the output feature of the first-level multi-scale wide-kernel convolution stem.

Subsequently, the MS-WDCNN branch adopts four one-dimensional convolutional blocks to further extract deep local features. Each convolutional block consists of a one-dimensional convolution, batch normalization, ReLU activation, and BlurPool1D downsampling. The output channels of the whole branch are 16, 32, 64, 64, and 64, respectively. Finally, the output of the one-dimensional convolution branch is obtained as follows:

(15) $$F_{c}\in R^{64\times L_{c}}$$

where $F_{c}$ denotes the one-dimensional local fault feature extracted by the MS-WDCNN branch.

### 3.3. Swin Transformer Branch

To model contextual dependencies in the weighted differential time–frequency features, this article constructs a single-stage Swin Transformer branch. Since the differential and spectral channels in WDF have been standardized and aligned to the same length, they can be reconstructed into a two-dimensional feature map with a consistent layout, which facilitates subsequent attention-based contextual modeling and reduces the influence of numerical scale differences between the two channels. First, the input feature is reconstructed into a two-dimensional feature map:

(16) $$X_{s}=Reshape\left(X\right)\in R^{C\times 32\times 32}$$

where C denotes the number of input channels. Then, $X_{s}$ is mapped into the embedding space through patch embedding and fed into the Swin Transformer branch for contextual feature modeling.

This branch adopts a single-stage structure consisting of patch embedding, two consecutive Swin Transformer blocks, and LayerNorm. Here, the single-stage structure means that no patch-merging operation is introduced throughout the branch, and the resolution of the feature map remains unchanged during contextual modeling. The two consecutive Swin Transformer blocks are used to sequentially perform window-based attention modeling and shifted-window information interaction within this stage. Compared with the original multi-stage Swin Transformer, this design can reduce the loss of details caused by feature compression, making it more suitable for bearing diagnosis tasks with subtle fault features and limited sample sizes.

**Figure 5:**
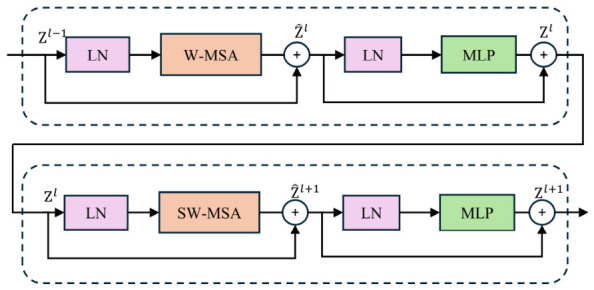
Structure of two consecutive Swin Transformer blocks.

In this article, the embedding dimension of this branch is set to 64, the depth is set to 2, the number of attention heads is set to 4, the window size is set to 4×4, and the MLP expansion ratio is set to 2. The structure of two consecutive Swin Transformer blocks is shown in Figure 5, and the corresponding computational process can be formulated as follows:

(17) $${\hat{z}}^{l}=W\text{-}MSA\left(LN\left(z^{l-1}\right)\right)+z^{l-1}$$

(18) $$z^{l}=MLP\left(LN\left({\hat{z}}^{l}\right)\right)+{\hat{z}}^{l}$$

(19) $${\hat{z}}^{l+1}=SW\text{-}MSA\left(LN\left(z^{l}\right)\right)+z^{l}$$

(20) $$z^{l+1}=MLP\left(LN\left({\hat{z}}^{l+1}\right)\right)+{\hat{z}}^{l+1}$$

where $z^{l}$ denotes the input feature of the l-th block, ${\hat{z}}^{l}$ denotes the intermediate feature after window-based attention, LN(⋅) denotes the LayerNorm operation, and MLP(⋅) denotes the multilayer perceptron. W-MSA and SW-MSA denote window-based multi-head self-attention and shifted-window multi-head self-attention, respectively.

After passing through the two Swin Transformer blocks, the output feature is normalized by LayerNorm to obtain the two-dimensional contextual feature representation:

(21) $$F_{s}\in R^{64\times H_{s}\times W_{s}}$$

where $F_{s}$ denotes the two-dimensional contextual feature extracted by the Swin Transformer branch.

### 3.4. Feature Interaction Module

**Figure 6:**
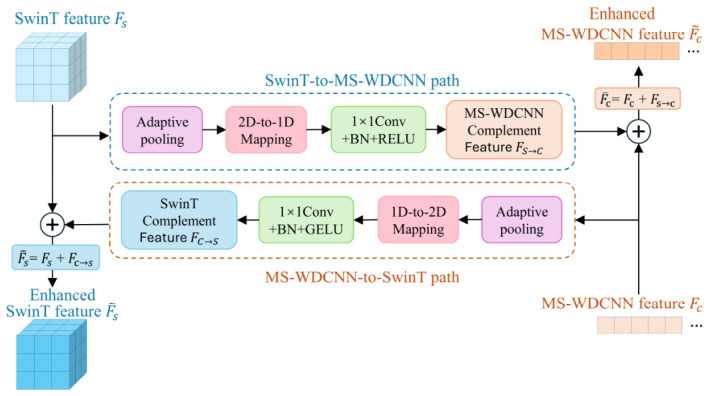
Structure of the proposed FIM.

Since the features extracted by the MS-WDCNN branch and the Swin Transformer branch differ in dimensional form and representation focus, direct fusion makes it difficult to fully utilize their complementary information. Therefore, this article designs a Feature Interaction Module (FIM) to establish bidirectional information transmission paths between the two branches. Through residual addition, cross-branch complementary information from the other branch is introduced while the original branch features are preserved, thereby enhancing the discriminability of the fused features. The structure of the FIM is shown in Figure 6. In this section, SwinT denotes the Swin Transformer branch used in this article.

To clearly describe the cross-branch dimensional alignment process in FIM, the MS-WDCNN branch feature and the SwinT branch feature are denoted with the batch dimension as follows:

(22) $$F_{c}\in R^{B\times C\times L_{c}}$$

(23) $$F_{s}\in R^{B\times C\times H_{s}\times W_{s}}$$

where B denotes the batch size; C denotes the number of channels, with C=64 in this article; $L_{c}$ denotes the length of the one-dimensional feature output by the MS-WDCNN branch; and $H_{s}$ and $W_{s}$ denote the spatial dimensions of the two-dimensional feature output by the SwinT branch. Since the two features have different spatial forms, the interaction mappings in FIM need to perform form conversion, scale alignment, and channel matching so that the generated supplementary features can be added to the corresponding target branch features through residual addition.

The FIM contains two interaction paths: SwinT-to-MS-WDCNN and MS-WDCNN-to-SwinT. To capture cross-branch complementary information at different scales, multi-scale adaptive average pooling is adopted in the two interaction paths, and the pooling-scale set is defined as follows:

(24) P={1,2,4}

where P denotes the set of adaptive average pooling scales.

In the SwinT-to-MS-WDCNN path, the two-dimensional SwinT feature $F_{s}$ is first processed by adaptive average pooling at different scales p∈P, producing a two-dimensional feature with the size of p×p. Then, the pooled two-dimensional feature is flattened into a one-dimensional sequence and aligned to the target length $L_{c}$ of the MS-WDCNN branch through one-dimensional interpolation. The aligned features obtained at different scales are averaged and then processed by a pointwise 1×1 Conv1D layer, followed by BatchNorm and ReLU activation to generate supplementary features for the MS-WDCNN branch:

(25) $$U_{s\to c}^{p}=I_{p^{2}\to L_{c}}\left(Flatten(AAP_{2D}^{p\times p}(F_{s}))\right)$$

(26) $$F_{s\to c}=\phi \left(BN\left(Conv_{1\times 1}^{1D}\left(\frac{1}{|P|}\sum_{p\in P}U_{s\to c}^{p}\right)\right)\right)$$

where $AAP_{2D}^{p\times p}(\cdot )$ denotes two-dimensional adaptive average pooling with an output size of p×p, Flatten(⋅) denotes the operation that flattens the two-dimensional feature into a one-dimensional sequence, $I_{p^{2}\to L_{c}}(\cdot )$ denotes one-dimensional interpolation from length $p^{2}$ to $L_{c}$, ∣P∣ denotes the number of pooling scales, and ϕ(⋅) denotes the ReLU activation function. After this path, the generated supplementary feature satisfies

(27) $$F_{s\to c}\in R^{B\times C\times L_{c}}$$

Therefore, $F_{s\to c}$ has the same dimensions as $F_{c}$ and can be added to it through a residual connection.

In the MS-WDCNN-to-SwinT path, the one-dimensional MS-WDCNN feature $F_{c}$ is first processed by adaptive average pooling at different scales p∈P, producing a one-dimensional feature with the length of $p^{2}$. Then, it is reconstructed into a two-dimensional feature map with the size of p×p and aligned to the target spatial size $H_{s}\times W_{s}$ of the SwinT branch through two-dimensional interpolation. The aligned features obtained at different scales are averaged and then processed by a 1×1 Conv2D layer, followed by BatchNorm and GELU activation to generate supplementary features for the SwinT branch:

(28) $$U_{c\to s}^{p}=I_{p\times p\to H_{s}\times W_{s}}\left(Reshape(AAP_{1D}^{p^{2}}(F_{c}))\right)$$

(29) $$F_{c\to s}=\psi \left(BN\left(Conv_{1\times 1}^{2D}\left(\frac{1}{|P|}\sum_{p\in P}U_{c\to s}^{p}\right)\right)\right)$$

where $AAP_{1D}^{p^{2}}(\cdot )$ denotes one-dimensional adaptive average pooling with an output length of $p^{2}$, Reshape(⋅) denotes the operation that reconstructs the one-dimensional feature into a two-dimensional feature map, $I_{p\times p\to H_{s}\times W_{s}}(\cdot )$ denotes two-dimensional interpolation from p×p to $H_{s}\times W_{s}$, and ψ(⋅) denotes the GELU activation function. After this path, the generated supplementary feature satisfies:

(30) $$F_{c\to s}\in R^{B\times C\times H_{s}\times W_{s}}$$

Therefore, $F_{c\to s}$ has the same dimensional form as the SwinT branch feature $F_{s}$ and can be used for subsequent residual addition.

Based on the above cross-branch mappings, the bidirectional interaction enhancement process can be expressed as follows:

(31) $${\tilde{F}}_{c}=F_{c}+F_{s\to c}$$

(32) $${\tilde{F}}_{s}=F_{s}+F_{c\to s}$$

where $F_{s\to c}$ denotes the supplementary feature generated from the SwinT branch for the MS-WDCNN branch, and $F_{c\to s}$ denotes the supplementary feature generated from the MS-WDCNN branch for the SwinT branch. ${\tilde{F}}_{c}$ and ${\tilde{F}}_{s}$ denote the interaction-enhanced MS-WDCNN branch feature and SwinT branch feature, respectively. Since the supplementary features are aligned to the dimensional forms of the corresponding target branch features before residual addition, this interaction mechanism can introduce cross-branch supplementary information while preserving the original branch features, thereby enhancing the complementary representation between local impulse features and contextual features.

After interaction enhancement by FIM, ${\tilde{F}}_{c}$ and ${\tilde{F}}_{s}$ are processed by one-dimensional global average pooling and two-dimensional global average pooling to obtain feature vectors:

(33) $$z_{c}=GAP_{1D}\left({\tilde{F}}_{c}\right)$$

(34) $$z_{s}=GAP_{2D}\left({\tilde{F}}_{s}\right)$$

where $z_{c}\in R^{64}$ and $z_{s}\in R^{64}$. Finally, the two feature vectors are concatenated to obtain the fused feature:

(35) $$z=\left[z_{c},z_{s}\right]\in R^{128}$$

which is then fed into a linear classifier for fault category identification.

## 4. Experimental Results and Analysis

The program was implemented in Python (version 3.11.14) using Visual Studio Code (version 1.127.0) as the development environment. The hardware platform was equipped with an Intel Core i7-12700H CPU, 16 GB RAM, and an NVIDIA GeForce RTX 3050 GPU (Santa Clara, CA, USA). All models were trained using the Adam optimizer, a learning rate of 0.0002, a batch size of 32, 50 training epochs, and the cross-entropy loss function.

To ensure fair comparisons, all models followed the same experimental protocol. For each repeated run, all models used the same training, validation, and test partitions and the same run-specific random seed. In the few-shot experiments, the same number of training samples per class and the same sampled training subsets were used for all models in each corresponding run, while the test set remained unchanged. The validation set was used only for model selection and performance monitoring during training, whereas the test set was used only for final performance evaluation. All experiments were repeated five times, and the mean accuracy over the five runs was reported as the final result. This run-wise correspondence provided the basis for paired statistical comparisons and ensured fair comparisons among different models.

### 4.1. Case I: CWRU Dataset

#### 4.1.1. Description of the CWRU Dataset

**Figure 7:**
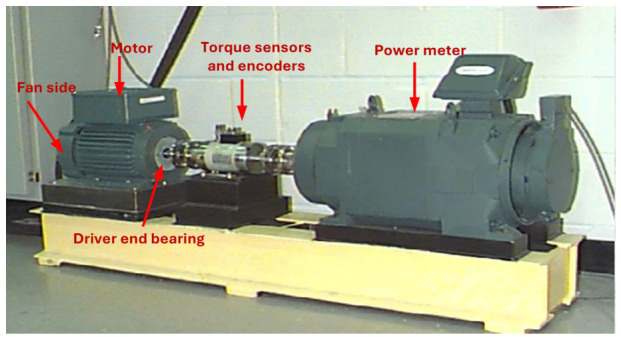
CWRU bearing test platform.

The public bearing fault dataset provided by the Case Western Reserve University Bearing Data Center was used for experimental validation [30], and the experimental platform is shown in Figure 7. The drive-end bearing vibration data sampled at 12 kHz were selected. The motor loads are 0, 1, 2, and 3 hp, corresponding to rotational speeds of 1797, 1772, 1750, and 1730 r/min, respectively. The dataset contains four health conditions: normal condition (Nor), inner race fault (IF), ball fault (BF), and outer race fault (OF). For the three fault types, three damage sizes of 0.007, 0.014, and 0.021 in were considered, resulting in 10 bearing health categories in total.

According to the different load conditions, the data were organized into four subsets, namely Load 0, Load 1, Load 2, and Load 3. To avoid data leakage, the original continuous vibration signals were first divided into training, validation, and test sets at a ratio of 7:2:1. Sliding-window sampling was then performed only within each divided data segment. The sample length was set to 1024, and the sliding step was set to 512. Finally, 220 samples were generated for each health category in each data subset, resulting in 2200 samples per data subset. The detailed distribution is listed in Table 1.

**Figure 8:**
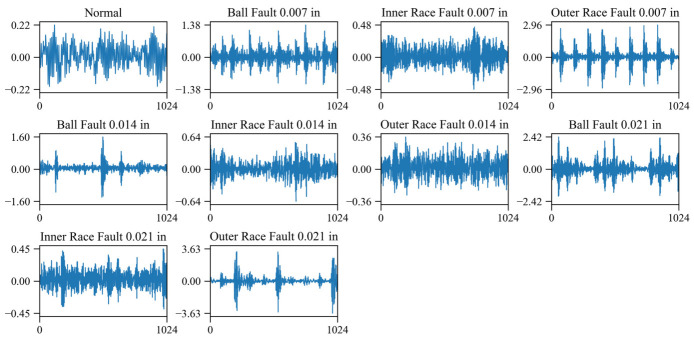
Time-domain waveforms of raw vibration signals.

After sample construction, time-domain waveform analysis is performed on the raw vibration samples, as shown in Figure 8. It can be observed that the impulse characteristics of some fault categories are not sufficiently obvious, and a single time-domain signal is difficult to fully represent complex fault information. Therefore, weighted differential time–frequency features (WDF) are further constructed as the model input, providing more discriminative fault representations for the subsequent dual-branch network.

To verify the effectiveness of the proposed model under variable operating conditions, eight diagnostic tasks are constructed based on the four load data subsets. The detailed task settings are listed in Table 2. Taking Task I as an example, Load 0 is used for training and validation, while the combined data from Load 1, Load 2, and Load 3 are used for testing.

#### 4.1.2. Diagnostic Results Under Variable Operating Conditions on the CWRU Dataset

**Figure 9:**
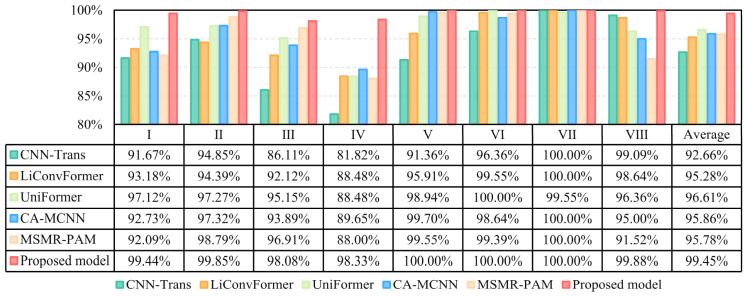
Comparison of diagnostic results under variable operating conditions on the CWRU dataset.

To verify the diagnostic performance of the proposed model under variable operating conditions, five representative deep learning models are selected for comparison, including CNN-Transformer (CNN-Trans), LiConvFormer [31], UniFormer [32], CA-MCNN [33], and MSMR-PAM [34]. All models are evaluated according to the task settings listed in Table 2. Each experiment is repeated five times, and the average accuracy is taken as the final result, as shown in Figure 9.

As shown in Figure 9, the proposed model achieved the highest average diagnostic accuracy of 99.45% across the eight tasks under variable operating conditions. The corresponding improvements over CNN-Trans, LiConvFormer, UniFormer, CA-MCNN, and MSMR-PAM were 6.79%, 4.17%, 2.84%, 3.59%, and 3.67%, respectively. In Task IV, all comparison models showed a clear performance decline, whereas the proposed model still maintained an accuracy of 98.33%. This indicates that the proposed model still has good generalization capability when there is a large distribution discrepancy between source and target operating conditions.

To further evaluate the statistical significance of the performance differences, a two-tailed paired t-test was conducted based on the results of five repeated runs. For each task, the proposed model was compared with the best-performing competitor in terms of average accuracy. As shown in Table 3, the proposed model achieved statistically significant improvements in Tasks I–V and Task VIII, with all p-values below 0.05. In Tasks VI and VII, both methods achieved an accuracy of 100.00% with no variation; therefore, the statistical significance test was not applicable.

Although CNN-Trans has the ability to extract local features and model global dependencies, its feature fusion mainly relies on simple concatenation and lacks an effective deep interaction mechanism. Therefore, its performance fluctuates significantly in complex tasks under variable operating conditions. UniFormer integrates local and global information through progressive fusion, but its feature integration process is still mainly based on serial modeling and lacks a multi-level complementary fusion mechanism. LiConvFormer, CA-MCNN, and MSMR-PAM all introduce multi-scale feature extraction structures, which can enhance the representation ability of fault features at different scales. However, their overall feature transmission still follows a relatively single-path manner, which is insufficient for comprehensively representing multiple types of fault information under complex variable operating conditions. In contrast, the proposed model extracts local fault impulse features and window-based contextual information through the MS-WDCNN branch and the Swin Transformer branch, respectively, and uses FIM to achieve bidirectional complementary fusion. The experimental results show that this structure can alleviate the insufficient feature representation problem of single-path methods in complex tasks under variable operating conditions, verifying the effectiveness of the dual-branch interactive fusion strategy.

#### 4.1.3. Robustness Analysis

**Figure 10:**
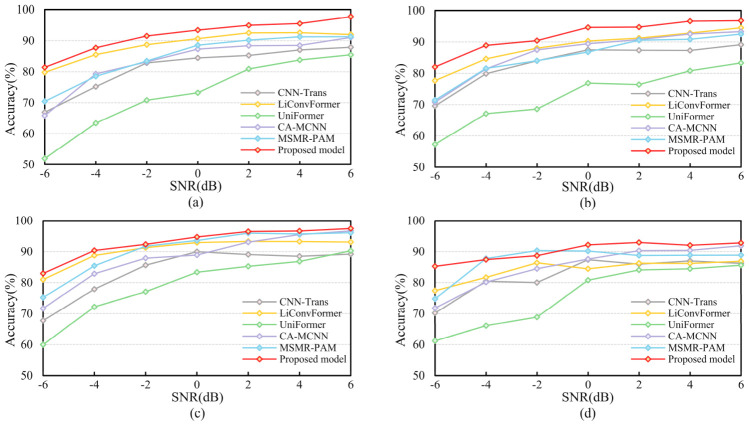
Comparison of noise robustness under variable operating conditions. (**a**) Task I; (**b**) Task II; (**c**) Task III; (**d**) Task IV.

To evaluate model robustness under noise interference, Gaussian white noise was added to Tasks I–IV at SNR levels of −6, −4, −2, 0, 2, 4, and 6 dB. The average diagnostic accuracy was used as the evaluation metric, as shown in Figure 10.

As shown in Figure 10, as the SNR increases from −6 dB to 6 dB, the diagnostic accuracy of all models generally shows an upward trend. However, different models exhibit clear differences in their adaptability to noise interference. UniFormer shows relatively low overall accuracy and large fluctuations, while the other four comparison models improve to some extent with increasing SNR but still suffer from obvious performance degradation under low-SNR conditions. In contrast, the proposed model consistently maintains the highest or near-highest accuracy across the four subtasks, and its performance changes more smoothly with SNR. In particular, under low-SNR conditions, the proposed model still maintains high recognition accuracy, indicating stronger robustness and generalization stability under the joint influence of variable operating conditions and noise interference.

#### 4.1.4. Results and Analysis Under Variable Operating Conditions and Few-Shot Settings on the CWRU Dataset

To further evaluate the diagnostic performance of the proposed model under few-shot settings and variable operating conditions, the more challenging Tasks I–IV were selected for few-shot experiments. For each task, 5, 7, 10, 15, and 20 samples from each health condition were randomly selected from the original training set and the original validation set for model training and validation, respectively. The test set remained unchanged to ensure the comparability of experimental results under different few-shot settings.

**Figure 11:**
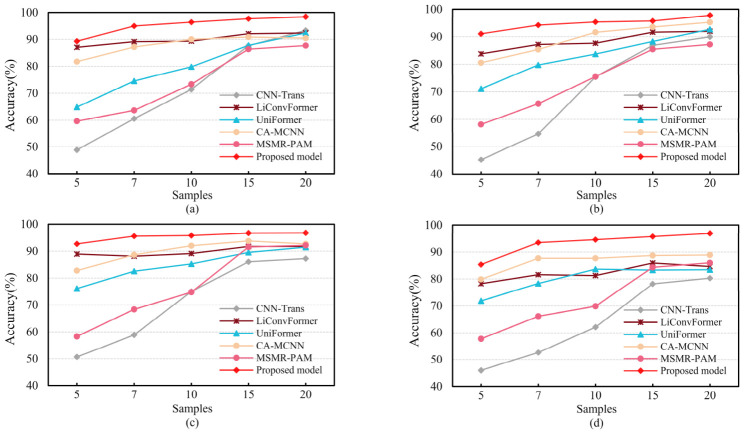
Comparison of diagnostic results under variable operating conditions and few-shot settings on the CWRU dataset: (**a**) Task I; (**b**) Task II; (**c**) Task III; (**d**) Task IV.

As shown in Figure 11, the diagnostic accuracies of all models generally increased as the number of training samples per class increased from 5 to 20. This indicates that increasing the number of available training samples improves the models’ ability to learn fault features. However, under limited-sample conditions, the performance differences among the comparison models were more pronounced. In particular, CNN-Trans and MSMR-PAM showed clear accuracy declines in some tasks, indicating insufficient feature-representation stability. By contrast, the proposed model maintained the highest diagnostic accuracy across all few-shot settings in the four tasks. Even under the most challenging five-shot setting, the proposed model achieved an average accuracy of 89.61% across Tasks I–IV and still maintained good diagnostic performance. This result provides a quantitative reference for the performance of the proposed method under extremely few-shot conditions and further demonstrates its ability to learn discriminative fault features from limited training samples.

#### 4.1.5. Sensitivity Analysis of the Weighting Coefficient α in WDF

To verify the rationality of the weighting coefficient α for the differential feature in WDF, a sensitivity analysis was conducted on CWRU Tasks I–IV under the 10-shot setting. In the experiment, α was set to 0.25, 0.50, 0.75, and 1.00, respectively, while the remaining network architecture and training parameters were kept unchanged. The results are shown in Table 4.

As shown in Table 4, when α was set to 0.25, the average accuracy was 94.38%, indicating that the local transient impulse information was not sufficiently enhanced when the weight of the differential channel was relatively small. When α was increased to 0.50, the model achieved the highest Task I accuracy of 96.39% and the highest average accuracy of 95.40%. As α was further increased to 0.75 and 1.00, although the results in some individual tasks improved slightly, the average accuracy decreased to 95.35% and 95.28%, respectively. This indicates that excessive enhancement in the differential feature may weaken the complementary role of the frequency-domain feature. Therefore, α=0.50 is adopted as the weighting coefficient in WDF to achieve a better balance between local impulse information enhancement and frequency-domain discriminative information preservation.

#### 4.1.6. Sensitivity Analysis of Multi-Scale Convolutional Kernel Sizes

The size of multi-scale convolution kernels affects the feature extraction capability of the MS-WDCNN branch. Therefore, a sensitivity analysis of multi-scale convolutional kernel sizes was conducted on Tasks I–IV to evaluate the influence of different kernel combinations on diagnostic performance.

As shown in Table 5, the proposed kernel combination k=63, 15, and $3\left(d=4\right)$ achieved the highest average accuracy of 98.92% and the best performance in Tasks I, II, and IV. These results indicate that the proposed combination provides a more balanced receptive-field configuration and enhances the feature-extraction capability of the MS-WDCNN branch under variable operating conditions.

#### 4.1.7. Comparative Analysis of Fusion Strategies on the CWRU Dataset

**Figure 12:**
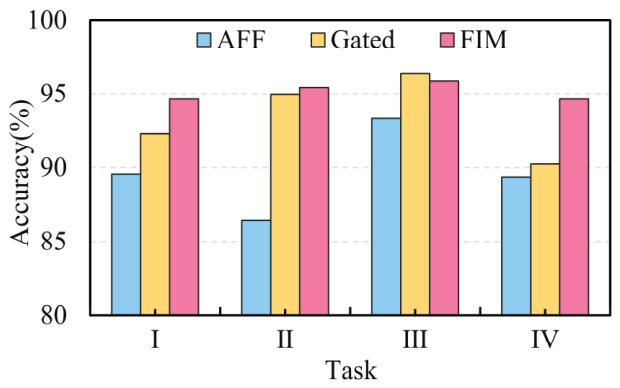
Comparison of results with different fusion strategies on the CWRU Dataset.

To further verify the effectiveness of the bidirectional interactive fusion design in the proposed model, attention feature fusion (AFF) and gated fusion were selected as comparison methods. AFF represents an attention-weighted feature fusion strategy, whereas gated fusion adaptively adjusts the contributions of different branch features through a gating mechanism. In the experiments, the remaining model architecture and training settings were kept unchanged, and only the fusion module was replaced. The comparison was conducted on Tasks I–IV with 10 training samples per class. The results are shown in Figure 12.

As shown in Figure 12, the proposed FIM achieved the highest accuracy in Tasks I, II, and IV. Although gated fusion slightly outperformed FIM in Task III, FIM still showed better overall diagnostic performance across the four tasks. Compared with AFF and gated fusion, the proposed FIM showed more balanced performance across different tasks. These results further demonstrate the effectiveness of the proposed FIM-based interaction strategy for few-shot diagnosis under variable operating conditions.

#### 4.1.8. Model Complexity and Computational Cost Analysis

The complexity and computational cost of each model on Task I are summarized in Table 6. The proposed model has only 0.11 M parameters, the fewest among all models, indicating a compact architecture and high parameter efficiency. However, the dual-branch feature extraction structure and FIM-based bidirectional interaction result in higher FLOPs, inference time, and training time than those of some lightweight comparison models. Together with the diagnostic results, these findings indicate that the proposed model achieves a favorable trade-off among parameter efficiency, computational cost, and diagnostic performance.

#### 4.1.9. Ablation Experiment on the CWRU Dataset

To verify the effectiveness of the key modules in the proposed model, ablation experiments were conducted on Tasks I–IV under both the full-sample setting and the setting with 10 training samples per class. The ablation models were constructed from two aspects: front-end input features and back-end network structures. For the front-end ablation, the back-end network was kept unchanged, and only the input representation was replaced. For the back-end ablation, the complete WDF input was kept unchanged, and only the feature extraction and fusion structures were adjusted. The specific ablation model settings are as follows:

1. M1: FFT frequency-domain features are used as the input to evaluate the diagnostic performance of a single frequency-domain representation.
2. M2: Raw signals are combined with FFT features to evaluate the effect of raw time-domain information.
3. M3: Diff-Raw replaces Raw in M2 to demonstrate the effect of differential enhancement on local impulse features.
4. M4: The complete proposed model is used to demonstrate the effectiveness of the proposed WDF input and FIM-based dual-branch fusion.
5. M5: FIM is removed from M4 to demonstrate the contribution of the feature interaction module.
6. M6: Only the Swin Transformer branch is retained to evaluate the performance of the window-context modeling branch.
7. M7: Only the MS-WDCNN branch is retained to evaluate the performance of the local convolutional feature extraction branch.
8. M8: WDCNN replaces MS-WDCNN to demonstrate the effectiveness of the multi-scale wide-kernel convolution design.

Table 7 and Table 8 show the experimental results of different ablation models under the full-sample setting and the 10-shot setting, respectively. By comparing M1–M4, it can be found that the average accuracy of the model improves progressively as the front-end feature construction is gradually enhanced, indicating that the proposed weighted differential time–frequency features can improve the discriminability of the input information. The comparison between M7 and M8 shows that, compared with the WDCNN branch, the proposed MS-WDCNN is more effective in capturing multi-scale local fault features.

The experimental results further show that when only the Swin Transformer branch (M6) or the MS-WDCNN branch (M7) was retained, the overall performance was lower than that of the dual-branch model M5. Notably, M6 showed a clear performance decline under the 10-shot setting. This is mainly because window-based self-attention and the reconstructed two-dimensional representation alone may have difficulty learning stable fault-related dependencies from very limited training samples. In addition, without the MS-WDCNN branch, M6 lacks direct local impulse extraction capability and is less sensitive to short-duration fault impacts. Therefore, the Swin Transformer branch is more suitable as a contextual complementary branch than as an independent fault feature extractor under few-shot settings. These results demonstrate the necessity of combining local fault impulse extraction with contextual relationship modeling.

However, M5 lacks an explicit cross-branch interaction mechanism, limiting the effective utilization of complementary information from the two branches. In contrast, the complete model M4 introduces FIM to enable bidirectional information exchange before final fusion, resulting in a clear improvement in diagnostic performance. These results further demonstrate the necessity and effectiveness of designing a dedicated interaction module for dual-branch features.

### 4.2. Case II: HUST Dataset

#### 4.2.1. Description of the HUST Dataset

**Figure 13:**
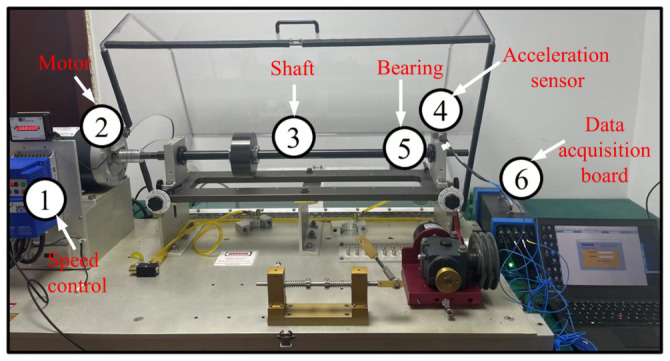
HUST bearing test platform.

Further experimental validation was conducted using the public dataset from the Spectra-Quest mechanical fault simulation test bench of Huazhong University of Science and Technology (HUST) [35]. The test platform is shown in Figure 13. The platform mainly consists of a speed controller, motor, transmission shaft, acceleration sensor, test bearing, and data acquisition board. The test bearing was ER-16K, and the vibration signals were collected by a three-axis acceleration sensor with a sampling frequency of 25.6 kHz. Three constant rotational speed conditions of 20, 25, and 30 Hz were selected, corresponding to 1200, 1500, and 1800 r/min, respectively. The dataset contains one normal condition and four fault conditions, including inner race fault, outer race fault, rolling element fault, and compound fault. Each fault type contains two damage levels, namely moderate and severe damage, resulting in nine bearing health conditions in total.

According to the different rotational speed conditions, the data were organized into three subsets, namely Dataset A, Dataset B, and Dataset C. To avoid data leakage, the original continuous signals in each data subset were first divided into training, validation, and test sets at a ratio of 7:2:1. Sliding-window sampling was then performed only within each divided data segment. The sample length was set to 1024, and the sliding step was set to 512. Finally, 250 samples were generated for each health condition, resulting in 2250 samples in each data subset. The detailed sample distribution is listed in Table 9.

After sample construction, the same processing method as that used for the CWRU dataset was adopted to convert the raw vibration samples into weighted differential time–frequency features (WDF), ensuring a consistent input form for the experiments on the two datasets.

To further verify the generalization performance of the proposed model under different rotational speed conditions, six diagnostic tasks under variable operating conditions with different training and testing rotational speeds are constructed based on the three rotational speed data subsets. The detailed task settings are listed in Table 10.

#### 4.2.2. Diagnostic Results Under Variable Operating Conditions on the HUST Dataset

**Figure 14:**
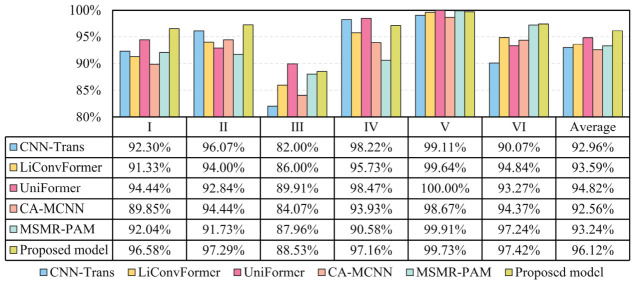
Comparison of diagnostic results under variable operating conditions on the HUST dataset.

To evaluate the generalization capability of the models under variable operating conditions and further examine the statistical reliability of the performance differences, all models were trained and tested according to the experimental tasks listed in Table 10. The results are shown in Figure 14 and Table 11.

As shown in Figure 14, the proposed model achieved an average accuracy of 96.12% across the six tasks under variable operating conditions. The corresponding improvements over CNN-Trans, LiConvFormer, UniFormer, CA-MCNN, and MSMR-PAM were 3.16%, 2.53%, 1.30%, 3.56%, and 2.88%, respectively, demonstrating the best overall performance. Although UniFormer achieved higher accuracies in Tasks III–V, the proposed model showed more balanced performance across the six tasks, maintaining accuracies above 96% in Tasks I, II, IV, V, and VI, with a relatively clear decline only in Task III. The results of the two-tailed paired t-test presented in Table 11 indicate that the performance differences between the proposed model and the best competitor in each task were statistically significant (p<0.05). Specifically, the proposed model significantly outperformed the corresponding best competitors in Tasks I, II, and VI, whereas UniFormer significantly outperformed the proposed model in Tasks III–V. Overall, although the proposed model did not achieve the highest accuracy in every task, it obtained the highest average accuracy and more balanced overall diagnostic performance.

#### 4.2.3. Results and Analysis Under Variable Operating Conditions and Few-Shot Settings on the HUST Dataset

**Figure 15:**
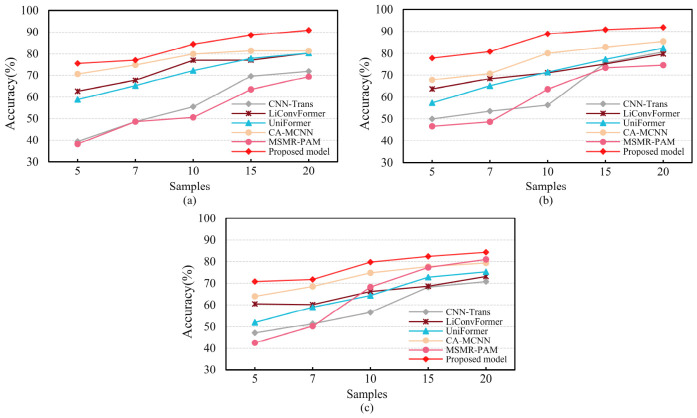
Comparison of diagnostic results under variable operating conditions and few-shot settings on the HUST dataset: (**a**) Task I; (**b**) Task II; (**c**) Task III.

To further evaluate the diagnostic performance of the proposed method under limited-sample conditions, the more challenging Tasks I–III were selected for the few-shot experiments. The experimental results are shown in Figure 15.

As shown in Figure 15, the proposed model maintained the best overall performance across the three tasks under different few-shot settings. Although the performance of most models improved as the number of training samples increased, the proposed model retained a clear advantage under the most challenging five-shot setting, in which only five training samples per class were available, and achieved an average accuracy of 74.73% across the three HUST tasks. These results further demonstrate its diagnostic effectiveness and generalization capability under variable operating conditions and few-shot settings.

#### 4.2.4. Comparative Analysis of Fusion Strategies on the HUST Dataset

**Figure 16:**
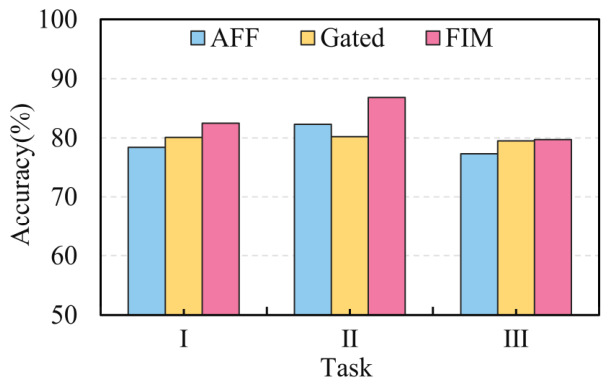
Comparison of results with different fusion strategies on the HUST Dataset.

To further verify the effectiveness of the bidirectional interactive fusion design in the proposed model, AFF and gated fusion were selected as comparison methods. Experiments were conducted on Tasks I–III with 10 training samples per class, and the results are shown in Figure 16.

As shown in Figure 16, the proposed FIM achieved the highest accuracy in all three tasks and showed better overall performance than AFF and gated fusion. These results indicate that the proposed bidirectional interaction mechanism more effectively utilizes the complementary information from the two branches under variable operating conditions and few-shot settings.

#### 4.2.5. Ablation Experiment on the HUST Dataset

To further verify the effectiveness of the key modules in the proposed model, eight ablation model variants, M1–M8, were constructed, and experiments were conducted on Tasks I–III under the setting of 10 training samples per class. All experiments were repeated five times, and the average accuracy was taken as the final result. The experimental results are shown in Table 12.

As shown in Table 12, by comparing M1–M4, the average accuracy increases from 77.47% to 85.22% as the front-end input features are gradually improved. This indicates that the proposed weighted differential time–frequency features can effectively enhance the discriminability of input signals and provide more effective information support for fault feature learning under few-shot settings. Compared with M5 without FIM, the average accuracy of the complete model M4 is improved by 5.23%, indicating that FIM can further promote information interaction and complementary fusion between the dual-branch features, thereby improving the few-shot diagnostic performance of the model under variable operating conditions.

## 5. Discussion

The performance decline on the HUST dataset is mainly attributable to its larger rotational-speed-domain shift and more complex fault distributions. Compared with CWRU, which covers a relatively narrow speed range, HUST includes rotational speeds of 1200, 1500, and 1800 r/min, resulting in greater variations in vibration-frequency components and fault-impulse distributions. In Task III, the model is trained on the highest-speed Dataset C and tested on the lower-speed Datasets A and B; therefore, the larger source–target speed discrepancy leads to a more pronounced performance decline. Nevertheless, the proposed method maintains favorable overall performance, demonstrating a certain degree of cross-speed generalization, although further improvement is still needed under larger speed discrepancies and complex compound-fault conditions.

Compared with global self-attention, the single-stage Swin Transformer branch performs contextual modeling through local window-based self-attention and shifted-window interaction. Global self-attention has quadratic computational complexity with respect to the number of tokens, whereas window-based attention scales approximately linearly with the number of tokens when the window size is fixed. This design reduces computational cost; however, the local-window constraint limits direct global-dependency modeling. Therefore, the Swin Transformer branch is used primarily for contextual modeling and is complemented by the local impulse features extracted by the MS-WDCNN branch.

From a deployment perspective, the dual-branch architecture and FIM-based bidirectional interaction introduce additional computational overhead despite the relatively small parameter count. The training time of approximately 140 s on the experimental platform suggests that frequent retraining or on-device adaptation may remain challenging on resource-constrained industrial controllers. At its current stage, the method is therefore better suited to offline training and subsequent inference deployment than to frequent on-device retraining. Future work will explore model pruning, lightweight interaction modules, and knowledge distillation to further reduce computational cost and support real-time online diagnosis.

FIM realizes bidirectional interaction between the MS-WDCNN and Swin Transformer branches through interpolation-based scale alignment and convolutional channel mapping. Because both branches currently output 64 channels, cross-branch dimensional matching is relatively straightforward. However, when the two branches have highly asymmetric channel capacities or substantially different sequence lengths and spatial resolutions, fixed channel projection and interpolation-based alignment may cause information compression or channel bottlenecks, thereby limiting cross-branch information transfer. Therefore, a principal algorithmic boundary of the current FIM lies in its channel-matching and scale-alignment capabilities under extremely asymmetric branch structures. Future work will investigate adaptive channel allocation, learnable channel projection, and dynamic scale weighting to improve the applicability of FIM to more complex heterogeneous network architectures.

## 6. Conclusions

This article proposes a bearing fault diagnosis method based on weighted differential time–frequency features and a dual-branch interactive fusion network to address insufficient single-feature representation and inadequate fusion between local fault impulse information and contextual information under variable operating conditions and few-shot settings. The effectiveness and advantages of the proposed method were validated on two public bearing datasets. The main conclusions are summarized as follows:

1. The weighted differential time–frequency features effectively enhance the fault discriminability of the input signals. WDF emphasizes local transient impulse information through first-order differencing and extracts FFT-based spectral amplitude information. Before channel stacking, the differential and spectral features are standardized to reduce inter-channel scale differences, and the spectral feature is aligned with the differential feature through linear interpolation to form a unified two-channel input representation. Compared with single FFT-based frequency-domain features and simply stacked time- and frequency-domain features, WDF improves the numerical comparability of the two feature streams and provides more discriminative input information, thereby improving diagnostic accuracy.
2. The dual-branch structure composed of MS-WDCNN and Swin Transformer simultaneously extracts local fault impulse features and window-based contextual information. The MS-WDCNN branch enhances local impulse feature extraction at different temporal scales through multi-scale wide-kernel convolution, while the Swin Transformer branch models contextual relationships in two-dimensional feature representations through window-based self-attention. Compared with the single-branch models, the dual-branch structure provides stronger feature representation and better diagnostic performance under variable operating conditions.
3. The proposed Feature Interaction Module promotes complementary fusion between the two branch features. Through bidirectional interaction, FIM enables more effective information exchange and complementarity between local impulse features and contextual features before final fusion. The ablation experiments and fusion-strategy comparisons show that introducing FIM further improves diagnostic performance, verifying the effectiveness of the proposed interactive fusion strategy.
4. In the experiments under variable operating conditions, the proposed method achieved average accuracies of 99.45% and 96.12% across the eight CWRU tasks and six HUST tasks, respectively. In the few-shot experiments, the proposed model also achieved the best overall performance among the compared models. These results demonstrate that the proposed method provides more balanced diagnostic performance and strong generalization under operating-condition variations and limited training samples.

Although the proposed method achieves favorable diagnostic performance, there is still room for further improvement in computational efficiency, cross-device adaptability, and real industrial online applications. The current experiments are mainly conducted on public bearing datasets with short-time sample segments, whereas continuous structural vibration noise, variable speed and load, sensor installation differences, and device-structure differences in real manufacturing scenarios may introduce more complex signal distribution variations. Future work will further improve the method from three aspects. First, model pruning, lightweight feature interaction modules, and knowledge distillation will be explored to reduce computational cost and improve the deployment efficiency of the model on edge devices. Second, cross-device validation under different equipment platforms, sensor arrangements, and sampling conditions will be conducted, and adaptive calibration or limited target-device sample updating will be considered to improve the adaptability of the model to multi-device scenarios. Finally, online diagnosis strategies for continuous vibration signal streams, including sliding-window detection, dynamic noise suppression, and model adaptive updating, will be investigated to further improve the practicality of the proposed method in intelligent operation and maintenance of real rotating machinery.

## Figures and Tables

**Table 1:** Sample Distribution of the CWRU Bearing Dataset.

Data Subset	Speed (rpm)	Fault Type/Label										Total
		Nor-/0	IF-7/1	IF-14/2	IF-21/3	BF-7/4	BF-14/5	BF-21/6	OF-7/7	OF-14/8	OF-21/9	
Load 0	1797	220	220	220	220	220	220	220	220	220	220	2200
Load 1	1772	220	220	220	220	220	220	220	220	220	220	2200
Load 2	1750	220	220	220	220	220	220	220	220	220	220	2200
Load 3	1730	220	220	220	220	220	220	220	220	220	220	2200

**Table 2:** Task Settings for Variable Operating Condition Experiments.

Task	Train Dataset/Number	Val Dataset/Number	Test Dataset/Number
I	Load 0/1540	Load 0/440	Load 1,2,3/660
II	Load 1/1540	Load 1/440	Load 0,2,3/660
III	Load 2/1540	Load 2/440	Load 0,1,3/660
IV	Load 3/1540	Load 3/440	Load 0,1,2/660
V	Load 1,2,3/4620	Load 1,2,3/1320	Load 0/220
VI	Load 0,2,3/4620	Load 0,2,3/1320	Load 1/220
VII	Load 0,1,3/4620	Load 0,1,3/1320	Load 2/220
VIII	Load 0,1,2/4620	Load 0,1,2/1320	Load 3/220

**Table 3:** Statistical Significance Test between the Proposed Model and the Best Competitor on the CWRU dataset.

Task	Best Competitor	Proposed Model (%)	Best Competitor (%)	*p*-Value
I	UniFormer	99.44 ± 0.23	97.12 ± 1.62	0.0378 *
II	MSMR-PAM	99.85 ± 0.14	98.79 ± 0.25	0.0110 *
III	MSMR-PAM	98.08 ± 0.63	96.91 ± 0.28	0.0098 *
IV	CA-MCNN	98.33 ± 0.16	89.65 ± 0.39	<0.0001 *
V	CA-MCNN	100.00 ± 0.00	99.70 ± 0.14	0.0093 *
VI	UniFormer	100.00 ± 0.00	100.00 ± 0.00	N/A
VII	LiConvFormer	100.00 ± 0.00	100.00 ± 0.00	N/A
VIII	CNN-Trans	99.88 ± 0.19	99.09 ± 0.40	0.0225 *

Notes: Values are reported as mean ± SD over five repeated runs. * indicates statistical significance at p<0.05 based on a two-tailed paired t-test. N/A indicates that the two methods produced identical results with zero variance.

**Table 4:** Diagnostic Performance with Different Weighting Coefficients α in WDF.

α	Accuracy/%				
	I	II	III	IV	Average
0.25	94.94	94.55	94.88	93.15	94.38
0.50	96.39	95.82	94.45	94.94	95.40
0.75	96.11	96.06	94.21	95.00	95.35
1.00	96.29	95.23	94.52	95.11	95.28

**Table 5:** Diagnostic Performance with Different Multi-Scale Convolutional Kernel Sizes.

Kernel Sizes	Accuracy/%				
	I	II	III	IV	Average
k = 63 only	97.88	99.24	98.48	95.91	97.88
k = 31, 15, 3	98.33	97.73	97.73	94.70	97.12
k = 63, 31, 15	98.79	97.50	96.87	91.82	96.24
k = 63, 15, 3	98.94	98.03	96.77	97.88	97.90
k = 63, 15, 3 (d = 4)	99.47	99.85	98.03	98.33	98.92

**Table 6:** Complexity and Computational Cost of Different Models.

Model	Params (M)	FLOPs (M)	Inference Time (ms)	Training Time (s)
CNN-Trans	0.21	39	2.4	24
LiConvFormer	0.57	51	4.6	50
UniFormer	1.78	42	3.4	37
CA-MCNN	1.88	146	5.7	87
MSMR-PAM	0.43	53	11.5	107
Proposed model	0.11	77	14.4	140

**Table 7:** Ablation Results under the Full-Sample Setting.

Model	Accuracy/%				
	I	II	III	IV	Average
M1	91.46	96.77	96.82	90.05	93.78
M2	98.64	98.08	96.62	90.86	96.05
M3	95.91	99.55	96.97	98.64	97.77
M4 (Proposed)	99.48	99.81	97.98	98.33	98.90
M5	99.09	98.48	98.48	93.18	97.31
M6	95.91	98.03	95.30	97.28	96.63
M7	90.64	97.12	94.39	89.64	92.95
M8	90.00	96.79	93.03	88.03	91.96

**Table 8:** Ablation Results with 10 Training Samples per Class on the CWRU dataset.

Model	Accuracy/%				
	I	II	III	IV	Average
M1	89.18	96.24	95.33	88.82	92.39
M2	97.33	90.70	95.24	91.42	93.67
M3	97.00	92.30	95.70	95.82	95.20
M4 (Proposed)	96.88	94.15	94.27	95.82	95.28
M5	94.91	95.18	96.12	91.64	94.46
M6	29.03	29.09	34.24	31.91	31.07
M7	89.61	89.70	89.52	85.94	88.69
M8	87.85	91.09	91.85	84.52	88.83

**Table 9:** Sample Distribution of the HUST Bearing Dataset.

Dataset	Speed (rpm)	Fault Type/Label									Total
		Nor/0	I-M/1	I-S/2	O-M/3	O-S/4	B-M/5	B-S/6	C-M/7	C-S/8	
Dataset A	1200	250	250	250	250	250	250	250	250	250	2250
Dataset B	1500	250	250	250	250	250	250	250	250	250	2250
Dataset C	1800	250	250	250	250	250	250	250	250	250	2250

**Table 10:** Task Settings for Variable Operating Condition Diagnosis.

Task	Train Dataset/Number	Val Dataset/Number	Test Dataset/Number
I	Dataset A/1575	Dataset A/450	Dataset B,C/450
II	Dataset B/1575	Dataset B/450	Dataset A,C/450
III	Dataset C/1575	Dataset C/450	Dataset A,B/450
IV	Dataset B,C/3150	Dataset B,C/900	Dataset A/225
V	Dataset A,C/3150	Dataset A,C/900	Dataset B/225
VI	Dataset A,B/3150	Dataset A,B/900	Dataset C/225

**Table 11:** Statistical Significance Test between the Proposed Model and the Best Competitor on the HUST dataset.

Task	Best Competitor	Proposed Model (%)	Best Competitor (%)	*p*-Value
I	UniFormer	96.58 ± 0.95	94.44 ± 0.67	0.0144 *
II	CNN-Trans	97.29 ± 0.58	96.07 ± 0.72	0.0079 *
III	UniFormer	88.53 ± 0.73	89.91 ± 0.62	0.0410 *
IV	UniFormer	97.16 ± 0.73	98.47 ± 0.67	0.0214 *
V	UniFormer	99.73 ± 0.21	100.00 ± 0.00	0.0440 *
VI	MSMR-PAM	97.42 ± 0.59	97.24 ± 0.53	0.0341 *

Note: Values are reported as mean ± SD over five repeated runs. * indicates statistical significance at p<0.05 based on a two-tailed paired t-test.

**Table 12:** Ablation Results with 10 Training Samples per Class on the HUST Dataset.

Model	Accuracy/%			
	I	II	III	Average
M1	75.87	84.04	72.49	77.47
M2	79.29	80.76	74.36	78.13
M3	85.00	88.11	81.31	84.81
M4 (Proposed)	85.31	89.38	80.98	85.22
M5	80.82	82.27	76.89	79.99
M6	26.36	26.89	24.58	25.94
M7	84.53	85.98	80.89	83.80
M8	85.38	85.42	81.02	83.94

## Data Availability

The data used to support the findings of this study are publicly available from the Case Western Reserve University Bearing Data Center and the Huazhong University of Science and Technology bearing dataset. The processed data are available from the corresponding author upon reasonable request.
